# Optimal attention deep learning based in-vehicle intrusion detection and classification model on CAN messages

**DOI:** 10.1038/s41598-025-10637-3

**Published:** 2025-09-30

**Authors:** R. Saravanan, S. Balaji, M. Ganesan, M. Braveen, R. Srinivasa Perumal

**Affiliations:** 1 Department of Information Technology, Sri Manakula Vinayagar Engineering College, Puducherry, India; 2 Department of Computer Science and Engineering, Sri Manakula Vinayagar Engineering College, Puducherry, India; 3 School of Computer Science and Engineering, Vellore Institute of Technology, Chennai, Tamilnadu India

**Keywords:** Intrusion detection, Controller area network, Deep learning, RMSProp optimizer, In-vehicle network, Attention, Engineering, Mathematics and computing

## Abstract

Intrusion detection systems (IDS) have enormous significance to ensure the security of high-tech automobiles, especially those using the controller area network (CAN) bus for transmission between different electronic control units (ECUs). The CAN is a popular transmission protocol in automobiles, but it is vulnerable to a variety of attacks. To overcome these issues, several studies have investigated the use of IDS for the CAN bus. Researchers have been exploring safety issues of inter- and intra-vehicular transmission. In recent times, intrusion detection sensors are gained popularity despite how easily and effectively they can identify intrusion. Deep learning (DL) and machine learning (ML) algorithms have demonstrated their effectiveness for accurately and quickly identifying intrusions. However, DL approaches need vast quantities of data to accomplish superior outcomes which may be difficult in the case of CAN-based IDS. This manuscript presents an Optimal Attention Deep Learning based In-vehicle Intrusion Detection and Classification (OADL-IVIDC) model to secure CAN messages in model vehicles. The model begins with data preprocessing phase to effectively transform the input data into a more suitable format. For in-vehicle IDS, the OADL-IVIDC framework employs an attention-based augmented long short-term memory (A-LSTM) model. To further optimize the performance of the OADL-IVIDC system, hyperparameters are adjusted using the root mean square propagation (RMSProp) algorithm. The performance of the OADL-IVIDC system is assessed using a standardized car hacking dataset. Experimental results demonstrate that the OADL-IVIDC approach outperforms other techniques in various performance measures.

## Introduction

As a key component of vehicle-to-everything (V2X) communication, Intelligent Connected Vehicles (ICVs) have rapidly gained prominence due to their integration with sophisticated in-vehicle communication systems and numerous embedded computing devices^1^. Besides many intelligent technologies combined into vehicles, ICVs can get numerous intellectual services and at-ease experiences, with collision avoidance, auto parking assist, autonomous driving, and much more^2^. However, the increased connectivity and technological complexity of ICVs also introduce a broader attack surface, exposing the in-vehicle network (IVN) to various cyber threats. A growing number of novel attacks are being developed in cyberspace, which directly compromise the security of IVNs and endanger passenger safety^3^. In current in-vehicle communication systems, the Controller Area Network (CAN) protocol is widely adopted as the primary communication standard among Electronic Control Units (ECUs)^4^. Despite its widespread use, the CAN protocol presents inherent security vulnerabilities due to its broadcast nature. Each CAN message contains only a message identifier and a set of control fields, but lacks essential security mechanisms such as sender authentication, source/destination addressing, or message integrity checks^5^. This allows attackers to inject malicious messages that may override legitimate instructions, potentially taking control of critical vehicle functions such as braking systems, powertrain components, or safety features–posing significant safety risks to both passengers and surrounding traffic^6^.

A vehicle is influenced by many kinds of attacks like Denial-Of-Service (DoS), replay, flooding, Sybil, routing, fuzzy, malfunction, impersonation, spoofing, proximity vulnerabilities, and remote sensor attacks^7^. Numerous researches have examined intra and inter-vehicular communications protection problems. For instance, intrusion detection sensors (IDS) have gotten huge attention because of how efficiently and effortlessly they can identify intrusions. The common present function of CAN protocol safety has concentrated on physical features such as restraining access control and encoding communication of CAN^8^. On the other hand, it is very essential to progress a more effective IDS^9^. Certainly, the efficacy of CAN bus communication can be moderated owing to physical access restrictions^10^. To manage the issue of conventional communication systems, Machine learning (ML)-based IDS methods were used.

This manuscript presents the development of an Optimal Attention Deep Learning-based In-vehicle Intrusion Detection and Classification (OADL-IVIDC) system, specifically designed to analyze CAN messages. The primary goal of the OADL-IVIDC system is accurately and efficiently detect intrusions within CAN traffic. The approach begins with a data preprocessing phase that formats the input data for optimal analysis. For intrusion detection, the system employs an attention-enhanced Long Short-Term Memory (LSTM) model, which is capable of capturing temporal patterns in sequential data. To further enhance model performance, hyperparameter tuning is conducted using the Root Mean Square Propagation (RMSProp) optimization algorithm. The proposed method is thoroughly evaluated using a widely recognized car hacking benchmark dataset, demonstrating its effectiveness in detecting in-vehicle network attacks.

## Literature survey

The increasing deployment of Intelligent Connected Vehicles (ICVs) has significantly heightened concerns around in-vehicle cybersecurity, particularly within the Controller Area Network (CAN) bus, which remains the dominant communication protocol in automotive embedded systems. As a result, numerous intrusion detection systems (IDSs) have been proposed to mitigate potential attacks targeting CAN-based vehicle communication.

Kang et al.^10^ proposed a deep learning-based IDS models tailored to in-vehicle networks, using a deep neural network (DNN) architecture to detect anomalies in CAN traffic. The proposed method demonstrated promising accuracy, but the model struggled with high false positive rates and lacked the ability to model temporal dependencies inherent in sequential data. Lee et al.^1^ introduced OTIDS, a lightweight intrusion detection framework that utilizes the properties of remote frames in the CAN protocol. The method provides a low-resource solution but does not leverage any learning algorithms for adaptive detection and therefore lacks adaptability to evolving attack scenarios. Wu et al.^7^ proposed an entropy-based anomaly detection scheme using a sliding window over CAN messages. This method is computationally efficient but sensitive to the size of the time window and lacks adaptability to evolving attack patterns. Similarly, Han et al.^8^ developed an event-triggered, interval-based anomaly detection system capable of detecting irregular patterns with low latency, albeit with limited effectiveness against sophisticated or multi-vector attacks. In contrast, Appathurai et al.^2^ investigated hardware-level vulnerabilities by simulating impersonation inject/impersonate (I2) vulnerabilities on IoV systems using an FPGA-based model. The proposed work highlights realistic attack vectors, it does not propose an IDS solution. Park et al.^4^ focused on functional validation of electronic control units (ECUs) using a hardware-in-the-loop simulation (HILS) for a steer-by-wire system, emphasizing control design over cybersecurity.

Elkhail et al.^3^ provided an extensive overview of vehicular threats and defense mechanisms but did not experimentally validate detection strategies. Ring et al.^5^ assessed the cybersecurity resilience of automotive electrical and electronic (E/E) architectures, underlining the importance of architectural design in ensuring security. Lo et al.^13^ proposed a hybrid CNN–LSTM deep learning framework to learn spatial–temporal patterns in CAN data, improving detection performance at the cost of computational complexity. Xiao et al.^14^ introduced a graph-based feature extraction framework using Graph Node Attention Networks (GNAT), which demonstrated superior feature representation but incurred graph construction overhead. Ding et al.^12^ offered a lightweight neural network for real-time CAN intrusion detection suitable for embedded deployment, albeit with reduced performance in complex attack scenarios.

Zulkifli et al.^27^ introduced an LSTM-RNN-based intrusion detection approach for CAN traffic, showcasing the advantages of temporal sequence modeling in vehicular networks. However, the absence of an attention mechanism limited the model’s ability to emphasize critical time steps during detection. To overcome this, Ahmed and Sharma^28^ proposed a hybrid Attention-CNN-LSTM model that combined spatial and temporal feature extraction, achieving higher detection accuracy. Despite its effectiveness, the added CNN layers increased the model’s complexity and computational load. Chen et al.^29^ presented a lightweight Transformer-based IDS that leveraged parallel processing to deliver strong performance on large-scale CAN datasets. However, its dependence on large volumes of training data and higher resource requirements may hinder its suitability for embedded automotive environments. These advancements illustrate the growing adoption of deep learning and attention mechanisms in CAN-based IDS research.

Ji et al.^11^ focused on feature selection to improve classification performance in CAN injection attacks, but the model optimized feature relevance but lacked temporal context. Dong et al.^15^ developed a multi-observation Hidden Markov Model (HMM)-based IDS, effectively modeled sequential data but showed limitations in adapting to new attack vectors. Nabil et al.^16^ proposed a hybrid model combining artificial neural networks (ANN) with LightGBM to achieve better performance through ensemble learning, although it required careful feature engineering. Khan et al.^17^ presented a statistical feature-based model that is simple and interpretable but less capable of handling previously unseen attacks.

Bhati et al.^18^ in the comprehensive review focuses on ensemble learning frameworks for Intrusion Detection Systems (IDS) emphasizing how combining multiple models–such as bagging, boosting, and stacking techniques–can enhance detection accuracy and robustness against diverse cyber threats. The review also discussed critical challenges, including computational complexity, class imbalance in network traffic, and limited adaptability to evolving attack vectors. Building on this, Bhati et al.^19^ proposed a voting-based ensemble IDS that integrates the outputs of multiple base classifiers to boost detection reliability. The model achieved improved performance in terms of reducing false positives and false negatives compared to individual classifiers, validating the advantages of ensemble approaches. However, the evaluation was conducted using standard network intrusion datasets, lacking direct application to vehicular network scenarios such as CAN bus communications. In^20^, proposed an ensemble IDS combining AdaBoost, Random Forest, and Logistic Regression, demonstrating strong performance on general datasets but lacking specific evaluation in vehicular network environments.

Shit et al.^9^ developed a crowdsource-enabled vehicle localization system using AI fingerprinting. Although not directly related to IDS, this work showcases AI’s broader applications in enhancing transportation safety by enabling precise vehicle tracking through unique hardware signatures collected from multiple sources. Jia et al.^21^ explored the use of attention-based Long Short-Term Memory (LSTM) networks for 4D aircraft trajectory prediction. The system validated the effectiveness of attention mechanisms in capturing temporal dependencies in sequential data, which is crucial for improving predictive accuracy in complex dynamic systems, suggesting potential benefits for similar temporal modeling challenges in IDS.

Ahda et al.^23^ conducted a comparative study between RMSProp and Adam optimizers, two popular methods for training deep learning models. The RMSProp provides more stable convergence when training sequence-based models like LSTMs, supporting its selection in IDS frameworks that rely on temporal pattern learning. While many existing approaches show promise, they often fall short in one or more aspects–such as lack of attention mechanisms, poor generalization to new attacks, high resource consumption, or shallow learning strategies. The proposed OADL-IVIDC framework addresses these limitations by integrating a lightweight Attention-LSTM model with RMSProp-based hyperparameter tuning. This combination facilitates efficient learning of temporal patterns and relevant features, enabling robust and scalable intrusion detection within CAN-based vehicle environments. The summary of related IDS research, highlights the evolution of detection methodologies from traditional statistical methods to complex hybrid deep learning models is summarised in the Table 1.

**Table 1:** Literature review of IDS approaches in intelligent vehicle networks.

References	Aim	Method	Advantage	Limitation	Dataset
Park et al., 2005	To evaluate ECU for Steer-by-Wire system	HILS for SBW ECU simulation	Enables ECU testing before deployment	No cybersecurity or IDS analysis	HILS Simulation
Kang & Kang, 2016	IDS using deep neural networks for IVN	Deep neural network	One of the earliest DL-based CAN IDS	Prone to false positives	Custom CAN data
Lee et al., 2017	Design OTIDS using remote frame in CAN	Lightweight IDS model	Low-resource CAN anomaly detection	No DL or advanced ML	CAN bus testbed
Ring et al., 2018	Evaluate automotive E/E cybersecurity	Architectural security analysis	Hardware attack surface awareness	Not focused on IDS	Simulation models
Wu et al., 2018	Detect anomalies using entropy-based sliding window	Statistical entropy for IDS	Fast anomaly detection	Sensitive to window size	CAN traffic logs
Appathurai et al., 2019	Analyze I2 beaconing attacks in IoV	FPGA-based simulation	Hardware realism	No IDS or detection model	Simulated CAN data
Bhati et al., 2020	Review IDS techniques	IDS taxonomy & classification	Broad coverage of IDS approaches	No experimental evaluation	N/A
Bhati & Khari, 2020	Propose voting ensemble IDS	Ensemble of multiple classifiers	Combines multiple model strengths	Not tailored for CAN or IVNs	Benchmark intrusion datasets
Bhati et al., 2022	Ensemble IDS using AdaBoost, RF, LR	Combined ML classifiers	Good generalization, robustness	Applied to conventional networks	Benchmark datasets
Lo et al., 2022	Hybrid CNN+LSTM for IVN intrusion	Spatiotemporal learning	Captures CAN traffic dynamics	Model complexity	CAN logs
Han et al., 2021	Interval-based anomaly detection	Time-triggered event windows	Lightweight and fast	Less adaptable to dynamic attacks	CAN dataset
Elkhail et al., 2021	Survey on automotive security	Comprehensive review	Covers attack types & countermeasures	No IDS model proposed	N/A
Shit et al., 2021	AI fingerprinting for secure localization	ML-based vehicle positioning	Combines location & security	Not IVN-focused	IoV dataset
Ding et al., 2023	Lightweight NN for CAN IDS	Simple NN for embedded systems	Low overhead	Lower performance in complex attacks	CAN injection dataset
Xiao et al., 2024	GNAT-based feature extraction for IVN IDS	Graph-based attention model	High accuracy	Requires graph construction overhead	CAN logs
Ji et al., 2024	Feature selection and classification	ML IDS for injection attacks	Efficient feature use	Shallow model	CAN attack dataset
Dong et al., 2023	HMM-based CAN IDS	Multi-observation HMM	Effective temporal pattern modeling	Less robust to new attacks	CAN logs
Nabil et al., 2024	ANN + LightGBM IDS	Hybrid model	High detection rate	Manual tuning required	Simulated dataset
Khan et al., 2023	Statistical attack-based IDS	Attack pattern modeling	Simplicity and interpretability	Poor adaptability	CAN logs
Bari et al., 2023	ML comparison for CAN IDS	Comparative ML analysis	Benchmarking utility	No DL or hybrid focus	Car Hacking Dataset
Wang et al., 2023	Graph learning for CAN IDS	Multi-view graph-based analysis	Strong structural feature learning	High computational cost	CAN logs
Jia et al., 2022	Attention-LSTM for trajectory prediction	A-LSTM for 4D aircraft tracking	Validates A-LSTM concept	Not vehicle IDS	Aircraft data
Ahda et al., 2024	Compare RMSprop and Adam	Optimizer study in NMT	RMSProp insight	Not applied to IDS	Translation dataset
Zulkifli, M. et al., 2022	Detect anomalies in CAN traffic using sequential learning	LSTM-RNN	Captures temporal patterns in sequential CAN data	Lacks attention mechanism to prioritize important features	Car Hacking Dataset
Ahmed et.al., 2023	Improve CAN IDS accuracy with spatial-temporal feature extraction	Attention-CNN-LSTM	Integrates spatial and temporal patterns for better detection	Increased computational complexity due to CNN layers	Custom CAN dataset
Chen et al., 2023	Develop a lightweight, high-performance IDS using parallel modeling	Transformer-based IDS	Enables parallel processing with good accuracy on large-scale data	Requires large training datasets and high computational resources	BitBrain CAN Dataset

## Proposed method

**Fig. 1:**
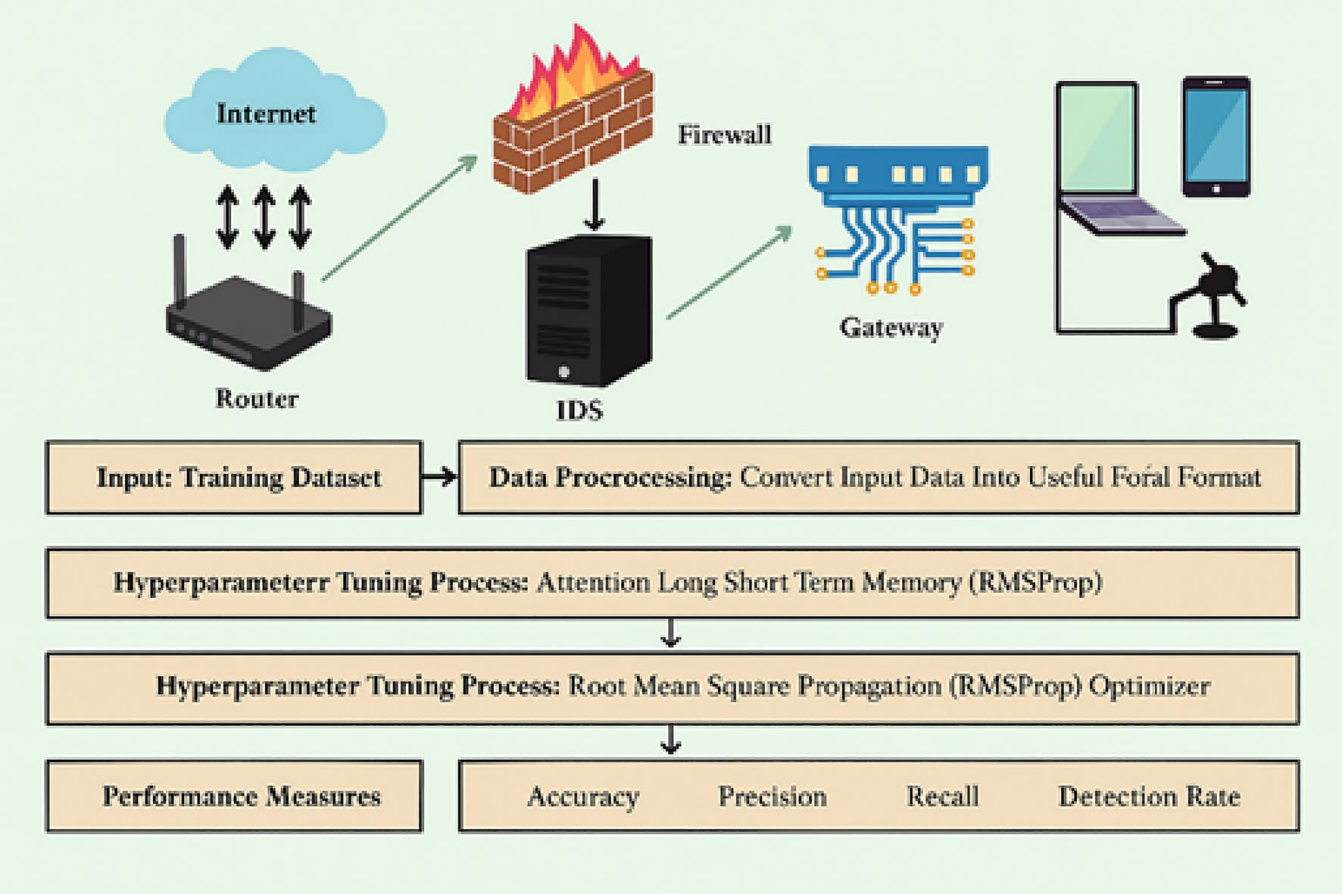
Workflow of OADL-IVIDC technique.

This paper presents the OADL-IVIDC strategy for analyzing CAN messages, aiming to accurately detect and classify intrusions within the vehicle network. The workflow of the proposed OADL-IVIDC model, as illustrated in Fig. 1, follows a structured sequence that includes data preprocessing, classification using the A-LSTM model, hyperparameter tuning via RMSProp optimization, and performance evaluation to ensure robust intrusion detection.

### Data pre-processing

The dataset used to evaluate the proposed OADL-IVIDC model is the publicly available Car Hacking Dataset, provided by the Hacking and Countermeasure Research Lab (HCRL) at Korea University. The dataset contains raw CAN bus traffic collected from a real 2010 Hyundai Sonata vehicle under normal and attack conditions. It includes over 1.3 million messages, covering one normal class and five attack categories: DoS, Fuzzy, Gear, RPM, and Spoofing attacks. As the initial step in the OADL-IVIDC pipeline, data preprocessing is crucial to ensure the CAN messages are structured for deep learning analysis. During preprocessing, missing values are checked and resolved to maintain data consistency. Attack messages are labeled to enable supervised classification. The dataset is then normalized using Standard Scaler to ensure uniform feature scaling, which aids in improving model convergence during training. Temporal features are preserved by dividing the data into 3-second windows, capturing sequential patterns relevant to intrusion detection. Table 2 summarizes the class distribution within the dataset, highlighting the percentage and count of samples in each category.

**Table 2:** Distribution of classes in the car hacking dataset.

Class label	Description	Number of samples	Percentage (%)
Normal	Benign CAN messages	1,056,000	80
DoS attack	DoS injection	153,600	11.6
Fuzzy attack	Random data fuzzing	76,800	5.8
Gear spoofing attack	False gear shift signals	19,200	1.4
RPM spoofing attack	False engine RPM signals	7680	0.6
Steering spoofing attack	False steering input signals	6400	0.6

The structured and preprocessed dataset is subsequently fed into the A-LSTM model, forming the basis for optimized classification and intrusion detection in the OADL-IVIDC framework.

### Classification using A-LSTM model

For in-vehicle intrusion detection, the OADL-IVIDC technique leverages the A-LSTM model to effectively manage the complexity of long sequential data. This is particularly beneficial in domains where Recurrent Neural Networks (RNNs) are commonly applied, and where recent advancements in deep learning have significantly improved sequence modeling capabilities^18^. However, traditional RNNs often suffer from issues such as vanishing or exploding gradients, which can hinder the learning process and reduce model performance on long-term dependencies. This issue is effectively addressed by the Long Short-Term Memory Neural Network (LSTM-NN), a specialized type of neural network designed to handle sequential data. LSTM networks consist of memory cells equipped with gating mechanisms that regulate the flow of information, enabling the model to retain important features across time steps and update its state based on the current input. LSTMs are particularly well-suited for time-series forecasting and are widely applied in domains like trajectory prediction due to their strong ability to capture long-term dependencies.

LSTM networks are defined by their distinctive gating mechanisms, including the input gate, forget gate, and output gate. In this architecture, $h_t$ represents the hidden state (or output) at time step t, $X_t$ is the input at that time, and $h_{t-1}$ denotes the hidden state from the previous time step. The input gate, denoted as $i_t$, regulates the extent to which new information is allowed into the cell state. The gate’s behavior is governed by a weight matrix $W_i$, and it is typically computed using the tanh activation function applied to a weighted combination of $x_t$ and $h_{t-1}$, along with a bias term. This activation helps determine how much of the current input should influence the memory cell. The specific mathematical formulation is presented in Eq. (1).

1 $$\begin{aligned} i_t=\sigma (W_i*[h_{t-1}X_t]+b_i ) \end{aligned}$$

In a similar manner, the output gate ot determines the portion of the cell state that contributes to the final output at time t. It is influenced by the previous hidden state $h_{t-1}$ and the current input $x_t$, both of which are combined through a weighted summation using the output gate’s weight matrix $W_o$, followed by the addition of a bias term. The result is then passed through the tanh activation function to regulate the output flow from the memory cell. This operation updates the output gate activation, as described mathematically in Eq. (2).

2 $$\begin{aligned} o_t=\sigma (W_0*[h_{t-1}X_t]+b_0) \end{aligned}$$

In the forget gate, the weight matrix $W_f$ directs the influence of both the previous hidden state $h_{t-1}$ and the current input xt on the gate’s activation. The forget gate output, denoted by ft, is computed by applying the sigmoid ($\sigma$) activation function to the weighted sum of $h_{t-1}$, $x_t$ and a bias term. This results in a value between 0 and 1, indicating the degree to which information from the previous cell state should be retained or discarded. A value closer to 1 implies that the information is preserved, while a value near 0 indicates it is forgotten. This gating mechanism is formally described in Eq. (3).

3 $$\begin{aligned} f_t=\sigma (W_f*[h_{t-1}X_t]+b_f) \end{aligned}$$

During the memory unit, $C_t$ represents the memory cell in time t. $i_t$ is multiplying by $\hat{C}_t$ and $f_t$ can be increased by the $C_{t-1}$ previously the two will be added for measuring $C_t$. The mathematical calculation form has been represented in Eq. (4). $W_C$ represents the memory cell’s weight matrix. The candidate cell layer $\hat{C}_t$ can multiply by the tanh of biasing and weighting $h_{t-1}$ and $x_t$. By employing the function of activation, $\hat{C}_t$ was acquired. The mathematical form can be represented in Eq. (5).

During the memory update phase, the cell state $C_t$ at time step t is computed by integrating both the retained information from the previous cell state and the new candidate values. Specifically, the forget gate output $f_t$ is element-wise multiplied with the previous cell state $C_{t-1}$ to preserve relevant historical information. Simultaneously, the input gate output $i_k$ is multiplied with the candidate cell state $C_t$, which is generated by applying a tanh activation to the weighted combination of the previous hidden state $h_{t-1}$ and the current input $x_t$. These two contributions are then added together to produce the updated cell state $C_t$, as expressed in Eq. (5), where, $W_C$ denotes the weight matrix used in computing the candidate cell state.

4 $$\begin{aligned} C_t=f_t*C_{t-1}+i_t*\hat{C}_t \end{aligned}$$

5 $$\begin{aligned} \hat{C}_t =\tanh (W_c*[h_{t-1},x_t ]+b_c ) \end{aligned}$$

Finally, the LSTM output at time step t, denoted as $h_t$, is derived by applying a tanh activation function to the updated cell state $C_t$, which is then element-wise multiplied by the output gate activation $o_t$. This operation regulates the extent of information exposed from the cell state to the hidden state, effectively determining the final output of the LSTM at that time step. The corresponding computation is represented in Eq. (6).

6 $$\begin{aligned} h_t=0_t*\tanh (C_t ) \end{aligned}$$

**Fig. 2:**
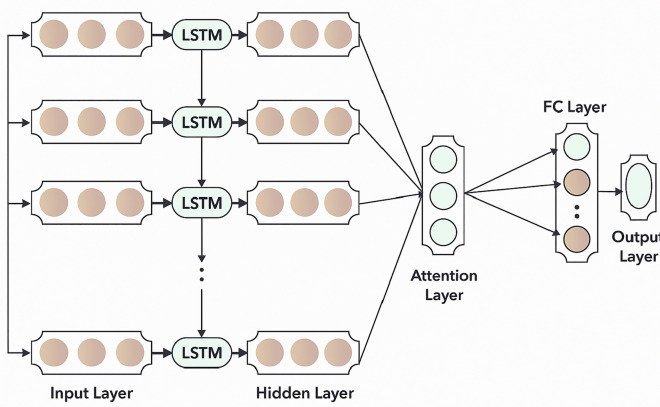
Structure of A-LSTM.

In CAN intrusion detection, the attention mechanism improves LSTM performance by highlighting the most relevant parts of sequential message data. Since CAN traffic includes time-based patterns–like specific ID or data byte sequences–attention helps the model prioritize anomalies such as sudden ID changes or irregular data injections during attacks (e.g., DoS, fuzzy, gear spoofing). This selective focus enables more accurate differentiation between normal and malicious activity. Additionally, attention assigns weights to each time step, offering clearer insights into which parts of the sequence influenced the model’s decision. This not only boosts detection accuracy but also improves interpretability–an essential feature in safety-critical automotive environments. By integrating the attention mechanism into DL models, the system is able to simulate a cognitive process similar to human reasoning–assigning higher importance to significant information and disregarding less relevant features. In the context of the attention module, the key parameters include the attention score $e_t$ and the context vector $C_t$. The attention score $e_t$ reflects the relative importance of different features at a given time step, and is calculated using Eq. (7).

7 $$\begin{aligned} e_t=\nu \tanh (W_e h_t+b_e ) \end{aligned}$$

Where $\nu$ and $W_e$ refers to the weight of the MLP while computing the attention weight, $b_e$ describes the bias, and $h_t$ defines the output of HL. $\sigma _t$ represents the attention weight concerning diverse characteristics in time t, and the evaluation form is Eq. (8).

8 $$\begin{aligned} \sigma _t=(expe_t)/\sum _{n}^{j=1} \end{aligned}$$

where, $e_j$ refers to the weight scores resultant for diverse features in time j. $C_t$ denotes the output in time t, and the computational model will be given in Eq. (9).

9 $$\begin{aligned} C_t=\sum _{n}^{j=1} \sigma _j h_j \end{aligned}$$

The attention mechanism is applied for computing and modifying the HL values concerning new output features, considers significant data, and completely learns and involves it, emphasizing the main parameter, and more consideration to the effect of the increase predictive accuracy, extracting internal connections, and predictive trajectory information. Figure 2 portrays the structure of A-LSTM.

### Hyperparameter tuning

To optimize the effectiveness of the proposed OADL-IVIDC framework, hyperparameter tuning is performed using the RMSProp optimization algorithm. RMSProp, a variant of stochastic gradient descent (SGD), dynamically adjusts the learning rate for each weight based on the running average of recent gradient magnitudes. This technique helps stabilize updates, particularly in deep networks, by preventing drastic learning rate fluctuations and enhancing convergence stability^19^. The RMSProp optimizer maintains a moving average of squared gradients using the following formulation:

10 $$\begin{aligned} r\leftarrow \rho r+(1-\rho )g^2 \end{aligned}$$

where, r denotes the moving average of squared gradients, $\rho$ is the decay rate (typically $\rho$ = 0.9), and g is the gradient of the current weight. The learning rate adjustment and parameter update are computed as:

11 $$\begin{aligned} \delta \theta = \frac{\epsilon }{ \sqrt{\delta + r}}g \end{aligned}$$

12 $$\begin{aligned} \theta \leftarrow \theta + \delta \theta \end{aligned}$$

where $\epsilon$ is the learning rate (set to 0.001), $\delta$ is a small constant (1e-8) to avoid division by zero, and $\theta$ represents the trainable model parameters.

The optimization process uses a fitness function (FF) to evaluate the performance of candidate hyperparameter combinations. In the OADL-IVIDC model, the fitness function emphasizes classification accuracy, particularly by maximizing the precision metric. The fitness score is calculated as:

13 $$\begin{aligned} Fitness = max (P) \end{aligned}$$

14 $$\begin{aligned} P=TP/(TP+FP) \end{aligned}$$

where TP and FP denote the number of true positives and false positives, respectively. The following hyperparameters in Table 3 were tuned and finalized for the A-LSTM classifier in the OADL-IVIDC pipeline:

**Table 3:** Distribution of classes in the car hacking dataset.

Hyperparameters	Value
Learning rate	0.001
Decay rate	0.9
Batch size	64
Epochs	100
LSTM hidden units	128
Number of layers	2
Dropout rate	0.3
Optimizer	RMSProp
Activation function	tanh (LSTM), softmax (output)
Sequence length	300 CAN messages

This optimization strategy enables the model to better generalize from training data and achieve higher detection accuracy for CAN intrusion instances.

**Algorithm 1:** OADL-IVIDC- optimized attention-based LSTM for in-vehicle intrusion detection.

**Input: ** C // Number of participating clients R // Total number of federated training rounds $$\epsilon$$ // Reconstruction error threshold $$X^t$$ // Local data at each client c $$\in$$ 1, 2, ..., C E // Local training epochs per round **Output:** Global model $$M^*$$, and Anomaly labels $$A^c$$ for each client // Training Phase Initialize global autoencoder model $$M^o$$ for each round r = 1 to R do Select subset $$S_r$$ $$\subseteq$$ 1, ..., C of clients for round r for each client c $$\in$$ $$S_r$$ (in parallel) do $$M^c \leftarrow M^{r-1}$$ // Receive current global model Train $$M^c$$ locally on $$X^c$$ for E epochs using autoencoder loss Send updated model weights $$W^c$$ to server end for Aggregate: $$M^r \leftarrow \sum (n^c / n_total) * W^c$$ using FedAvg end for $$M^* \leftarrow final global model M^R$$ // Inference Phase (Local) for each client c = 1 to C do $$A^c \leftarrow \emptyset$$ for each transaction $$x_i \in X^c$$ do $$z_i = fenc(x_i) = \sigma (Wx_i + b)$$ $$\hat{x}_i = fdec(z_i) = \sigma (Wz_i +b)$$ $$e_i = \vert \vert x_i - \hat{x}_i \vert \vert ^2$$ if $$e_i > \epsilon$$ then $$A^c \leftarrow A^c \cup$$ 1 // Anomaly else $$A^c \leftarrow A^c \cup$$ 0 // Normal end if end for end for return $$M^*, {A^1, A^2,..., A^c}$$	

The proposed OADL-IVIDC algorithm is designed to detect intrusions in vehicle CAN bus messages by using a deep learning model called A-LSTM, which combines Long Short-Term Memory (LSTM) with an attention mechanism to focus on important parts of the data. First, raw CAN data is collected and preprocessed to remove noise and format it for analysis. Then, the A-LSTM model is defined and trained, with its hyperparameters–like learning rate and batch size–optimized using the RMSProp optimizer, which helps the model learn more efficiently by adjusting how fast it updates. After training, the model classifies new messages as normal or anomalous. Finally, the performance is evaluated based on how accurately it detects intrusions

## Performance validation

This section presents the performance evaluation of the OADL-IVIDC technique using the Car Hacking dataset^22,24^. The dataset includes different types of attacks such as DoS, fuzzy attacks, spoofing of the drive gear, and spoofing of the RPM gauge. The data was collected by logging CAN traffic through the OBD-II port of a real vehicle during the execution of message injection attacks. Each dataset contains approximately 300 message injection intrusions, with each intrusion lasting for 3 to 5 seconds, while the total CAN traffic recorded spans 30 to 40 minutes.

1. DoS Attack: Involves injecting messages with ’0000’ CAN ID every 0.3 milliseconds, where ”0000” is the most frequent identifier.
2. Fuzzy Attack: Consists of injecting messages with random CAN IDs and data values every 0.5 milliseconds.
3. Spoofing Attack (RPM/Gear): Involves injecting messages with specific CAN IDs related to RPM or gear information every 1 millisecond.

**Fig. 3:**
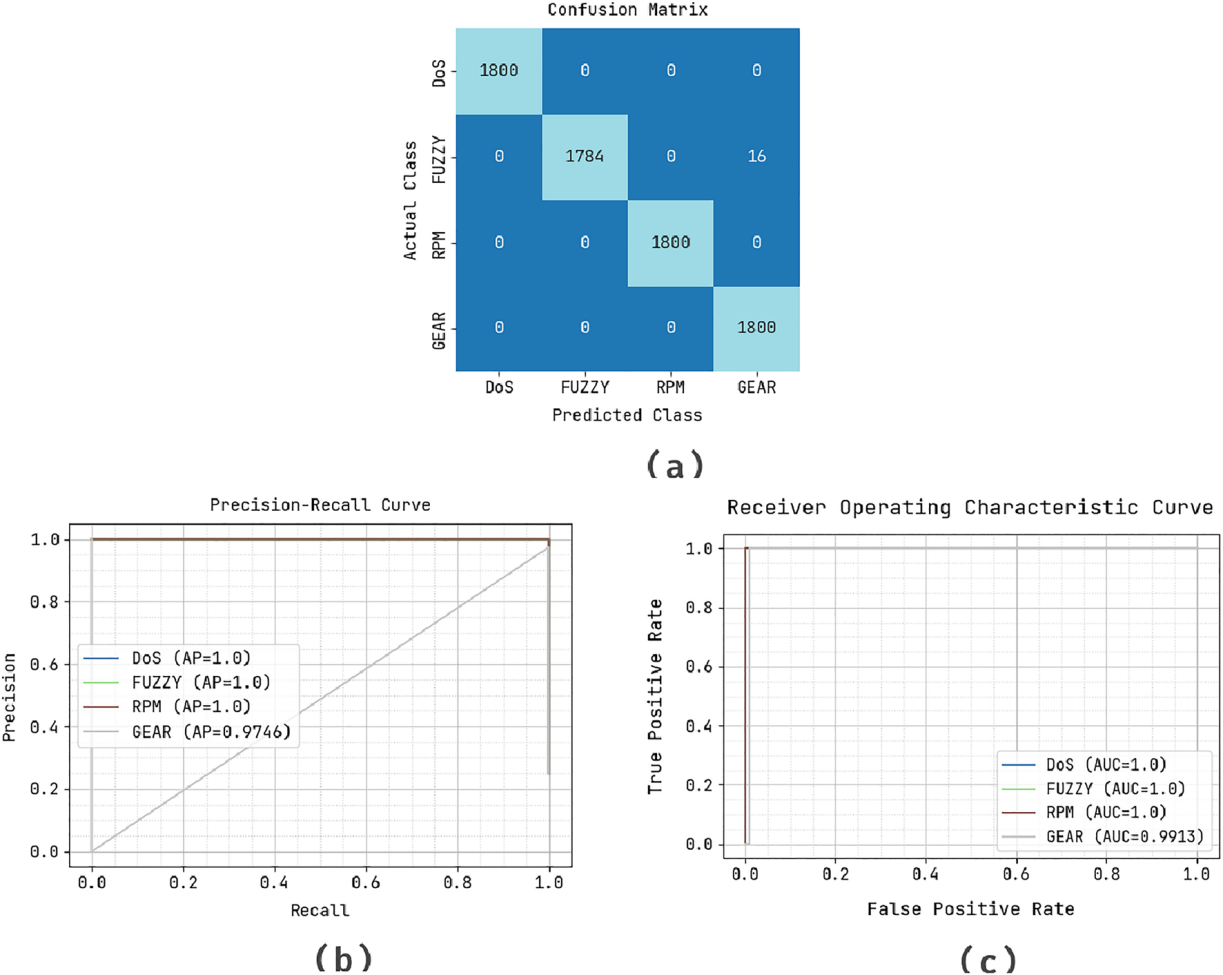
Classifier performance of (**a**) confusion matrix, (**b**) PR curve, and (**c**) ROC curve.

Figure 3 illustrates the classification results of the OADL-IVIDC system using the test dataset. In Fig. 3a , the confusion matrix demonstrates that the OADL-IVIDC technique accurately detected and classified all four class labels. The confusion matrix shows near-perfect classification accuracy. DoS, RPM, and GEAR attacks are fully detected with no misclassifications. FUZZY attack has 16 misclassifications (predicted as GEAR), indicating a slight overlap or similarity in patterns, but still an extremely high detection rate. This reflects high precision and recall for all classes, especially considering the complexity of CAN traffic. Furthermore, Fig. 3b highlights the precision-recall (PR) analysis of the OADL-IVIDC method, showing that it achieved the highest PR performance across all classes. Lastly, Fig. 3c provides the ROC analysis, revealing that the OADL-IVIDC approach produced excellent results with the highest ROC values across different class labels.

**Fig. 4:**
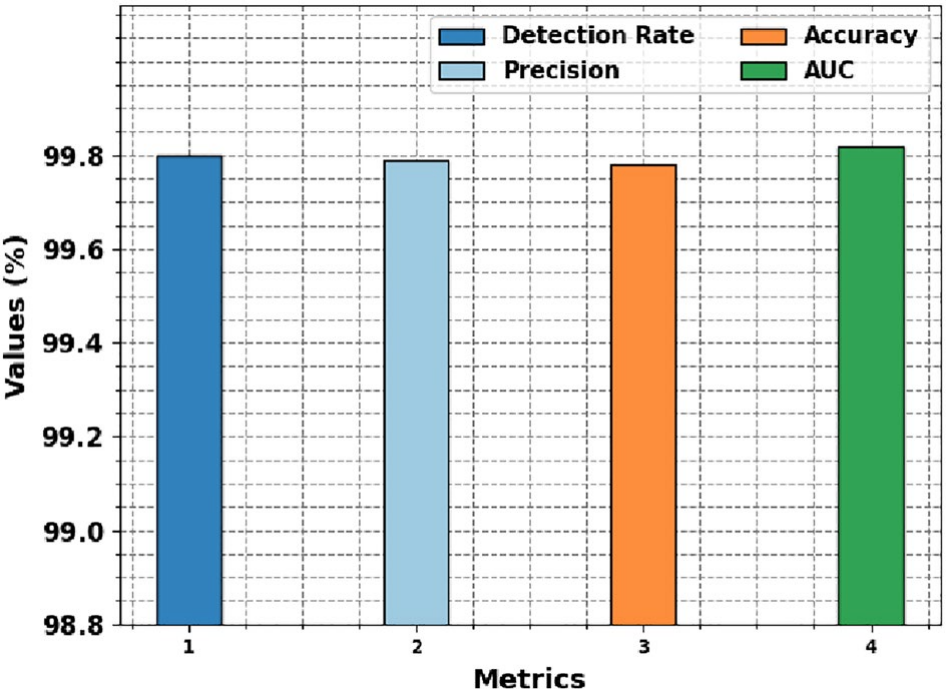
In-vehicle intrusion detection result of OADL-IVIDC technique with various measures.

Figure 4 and Table 4 signify a brief in-vehicle intrusion detection outcome of the OADL-IVIDC system. The experimentation values described that the OADL-IVIDC technique gets effectual classification of the intrusions. It is observed that the OADL-IVIDC approach gets a detection rate of 99.80%, precision of 99.79%, accuracy of 99.78%, and AUC of 99.82%.

**Table 4:** In-vehicle intrusion detection result of OADL-IVIDC technique with various measures.

Metrics	Values (%)
Detection rate	99.80
Precision	99.79
Accuracy	99.78
AUC	99.82

**Fig. 5:**
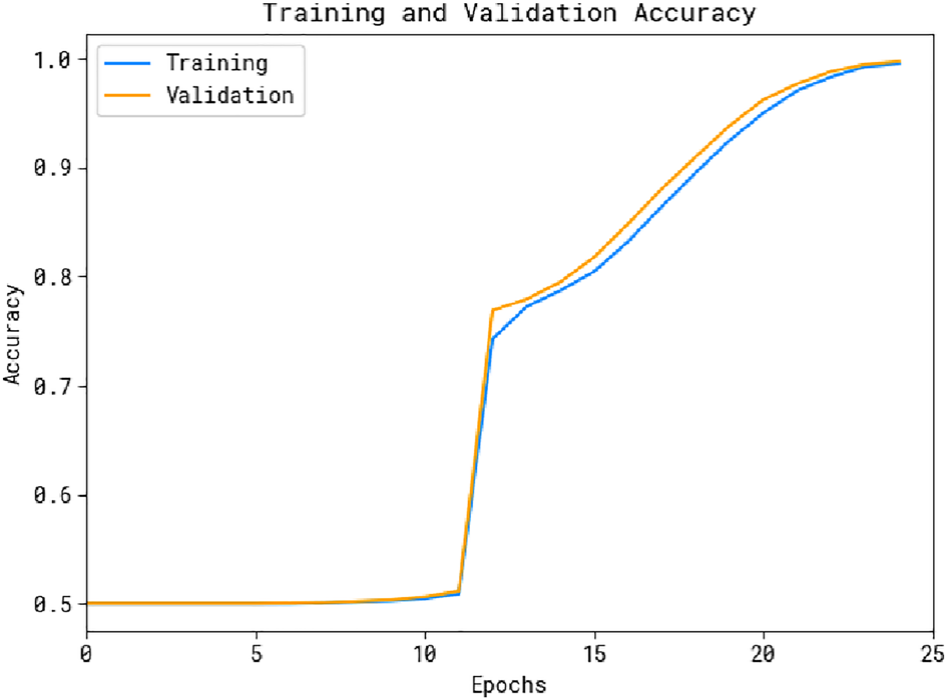
Accuracy curve of the OADL-IVIDC technique.

**Fig. 6:**
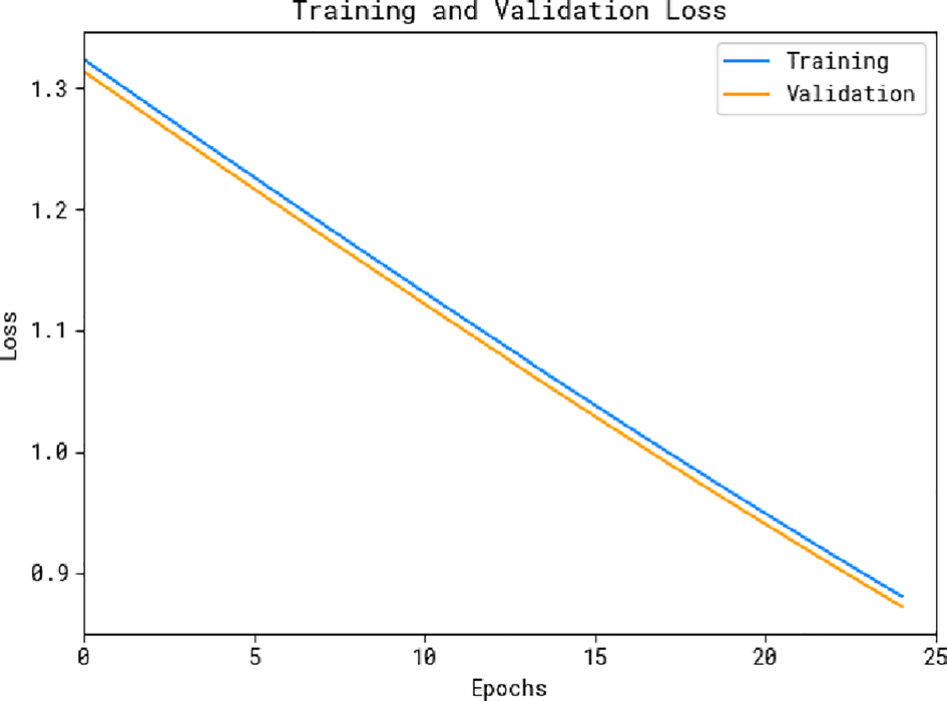
Loss curve of the OADL-IVIDC technique.

The performance of the OADL-IVIDC method is visually represented in Fig. 5 through validation accuracy and training accuracy curves. The figure provides a clear understanding of the OADL-IVIDC approach’s behavior across multiple epochs, highlighting its learning process and generalization capabilities. Notably, the figure indicates a steady improvement in both validation accuracy and training accuracy curves as the number of epochs increases. This demonstrates the adaptive nature of the OADL-IVIDC method in pattern recognition for both training and test data. The upward trend in validation accuracy reflects the technique’s ability to adapt to the training data and effectively identify previously unseen data, showcasing its strong generalization performance.

Figure 6 provides a detailed visualization of the training loss and validation loss results of the OADL-IVIDC system across various epochs. The gradual decrease in training loss demonstrates the system’s ability to refine weights and minimize classification errors on both training and test data. The figure offers a clear illustration of the OADL-IVIDC model’s relationship with the training data, emphasizing its capacity to identify patterns in both datasets. Notably, the OADL-IVIDC system consistently improves its parameters to reduce discrepancies between predicted and actual training classes.

**Fig. 7:**
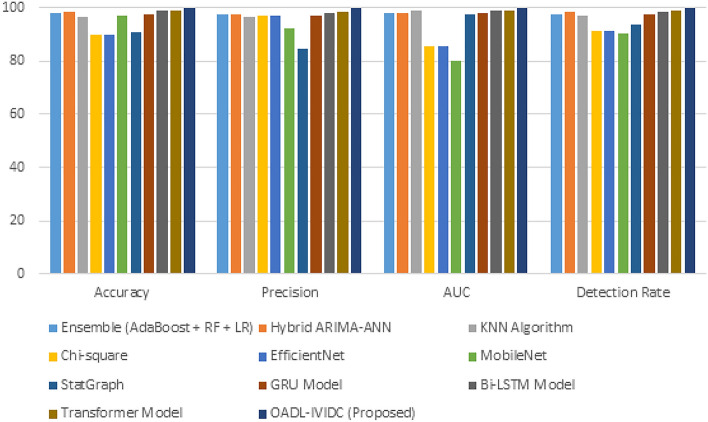
Comparative analysis of OADL-IVIDC technique with other algorithms.

The accuracy performance of the proposed OADL-IVIDC system is presented in Table 5 and illustrated in Fig. 7^24,25^. Using the same car hacking dataset and consistent training settings for all models, OADL-IVIDC achieved the highest accuracy of 99.78%. This surpasses traditional and deep learning methods, including Ensemble (AdaBoost + RF + LR) with 97.70%, Hybrid ARIMA-ANN (98.30%), KNN (96.40%), Chi-square (89.90%), EfficientNet (89.90%), MobileNet (96.88%), and StatGraph (90.50%). Among advanced deep learning models, Bi-LSTM and Transformer achieved notable accuracy scores of 98.70% and 98.95%, respectively, yet still fell short of the proposed method. These results confirm that OADL-IVIDC delivers the most accurate classification of CAN messages for in-vehicle intrusion detection.

In terms of precision, which measures the model’s ability to correctly classify only the relevant (true positive) instances, OADL-IVIDC again leads with a score of 99.79%. Other models recorded lower precision, including SVM (97.50%), KNN (96.40%), StatGraph (84.47%), and MobileNet (92.26%). Deep learning competitors like GRU (97.00%), Bi-LSTM (98.10%), and Transformer (98.50%) performed well but still did not exceed the precision achieved by OADL-IVIDC. This high precision indicates that the proposed model minimizes false positives and ensures reliable detection, a critical requirement in automotive safety applications.

The AUC metric evaluates the model’s ability to distinguish between attack and normal classes. OADL-IVIDC attained the highest AUC of 99.82%, indicating near-perfect classification capability. In contrast, the AUC values for traditional methods were lower–SVM (98.04%), Decision Tree (97.07%), Chi-square (85.60%), and MobileNet (80.15%). Among deep learning models, Transformer achieved 99.10%, Bi-LSTM 98.90%, and GRU 98.10%. Although Transformer came close, its higher computational demands make it less suitable for embedded applications. The superior AUC of OADL-IVIDC underscores its effectiveness in distinguishing intrusions across diverse CAN message patterns.

The Detection Rate (DR) reflects the model’s capability to correctly identify true attack instances. OADL-IVIDC recorded the highest DR at 99.80%, outperforming SVM (98.56%), Decision Tree (98.15%), KNN (97.02%), Chi-square and EfficientNet (91.00% each), MobileNet (90.18%), and StatGraph (93.47%). Among deep learning baselines, Bi-LSTM (98.40%), Transformer (98.70%), and GRU (97.30%) performed well, yet none matched the OADL-IVIDC’s ability to detect malicious CAN messages with such high reliability. This proves the robustness of the model in real-time detection scenarios.

The proposed OADL-IVIDC framework outperforms both conventional machine learning and recent deep learning approaches. Its integration of an attention-enhanced LSTM and RMSProp optimization ensures high classification performance, fast convergence, and low false alarms, making it a highly practical and scalable solution for real-time in-vehicle intrusion detection in intelligent connected vehicles.

**Table 5:** Comparative analysis of OADL-IVIDC technique with other algorithms.

ML techniques	Accuracy	Precision	AUC	Detection rate
Ensemble (AdaBoost + RF + LR)	97.70	97.50	97.90	97.60
Hybrid ARIMA-ANN	98.30	97.50	98.10	98.20
KNN algorithm	96.40	96.40	98.90	97.02
Chi-square	89.90	97.10	85.60	91.00
EfficientNet	89.90	97.10	85.60	91.00
MobileNet	96.88	92.26	80.15	90.18
StatGraph	90.50	84.47	97.25	93.47
GRU model	97.35	97.00	98.10	97.30
Bi-LSTM model	98.70	98.10	98.90	98.40
Transformer model	98.95	98.50	99.10	98.70
OADL-IVIDC (proposed)	99.78	99.79	99.82	99.80

The comparative performance of the proposed OADL-IVIDC system is presented in Table 5 and illustrated in Fig. 7 based on four key evaluation metrics: accuracy, precision, AUC (area under curve), and detection rate^25,26^. Using a consistent car hacking dataset and identical training conditions across all models, OADL-IVIDC demonstrated clear superiority over traditional machine learning techniques. In terms of accuracy, it achieved a leading score of 99.78%, outperforming SVM (97.50%), Decision Tree (99.40%), KNN (96.40%), Chi-Square (89.90%), EfficientNet (89.90%), MobileNet (96.88%), and StatGraph (90.50%). A similar trend is observed in precision, where OADL-IVIDC reached 99.79%, while the others ranged from 84.47% to 99.40%. Regarding AUC–an essential metric reflecting classification quality—OADL-IVIDC again led with 99.82%, surpassing KNN (98.90%), SVM (98.04%), and Decision Tree (97.07%), whereas lightweight models like MobileNet and EfficientNet lagged significantly with AUC scores of 80.15% and 85.60%, respectively. Further comparisons with deep learning models, including GRU, Bi-LSTM, and Transformer, confirmed the robustness of OADL-IVIDC. GRU offered better results than conventional methods due to its simplified gating, while Bi-LSTM improved performance by capturing bidirectional temporal dependencies. The Transformer achieved high AUC and accuracy but incurred heavy computational costs, limiting its feasibility for real-time in-vehicle applications. In contrast, OADL-IVIDC delivered the highest overall performance across all metrics, owing to its integration of a lightweight attention mechanism with LSTM for focused temporal learning, and the adoption of RMSProp optimization for stable convergence.

These features collectively make OADL-IVIDC not only the most accurate but also a computationally efficient and scalable solution for real-time CAN-based intrusion detection in intelligent vehicle systems. Furthermore, based on Detection Rate (DR), the OADL-IVIDC method acquires an improved DR of 99.80% whereas the SVM, DT, KNN, Chi-Square, EfficientNet, MobileNet, and StatGraph techniques acquire diminished DR values of 98.56%, 98.15%, 97.02%, 91%, 91%, 90.18%, and 93.47%, correspondingly. Thus, the OADL-IVIDC technique can be applied to the in-vehicle intrusion recognition process.

### Ablation study

To evaluate the contribution of each architectural and training component in the proposed OADL-IVIDC framework, an ablation study has been conducted using the car hacking dataset, which includes realistic in-vehicle CAN traffic under both normal and attack scenarios. Four model variants were tested, and their performance is summarized in Table 6. The baseline LSTM-only model, which uses a standard optimizer without attention, achieved an accuracy of 97.42%, precision of 96.95%, and AUC of 97.80%, indicating limited capability in capturing subtle intrusion patterns in sequential CAN messages. When the attention mechanism was added (LSTM + Attention), the model’s performance improved significantly–particularly in AUC (98.90%)—showing that attention helps the model prioritize anomalous temporal patterns. Similarly, substituting the standard optimizer with RMSProp (LSTM + RMSProp) led to noticeable gains (AUC: 98.60%), reflecting RMSProp’s effectiveness in managing learning dynamics on time-series data. The full OADL-IVIDC model (A-LSTM + RMSProp) outperformed all other variants with 99.78% accuracy, 99.79% precision, and 99.82% AUC, confirming that the combined use of attention and RMSProp optimization yields the best results for real-time, accurate intrusion detection on CAN traffic. These findings emphasize the importance of each component in enhancing the model’s performance for in-vehicle cybersecurity tasks.

**Table 6:** Ablation study results.

Model	Accuracy (%)	Precision (%)	AUC (%)
LSTM only	97.42	96.95	97.80
LSTM + attention	98.63	98.05	98.90
LSTM + RMSProp	98.26	97.84	98.60
A-LSTM + RMSProp (OADL-IVIDC)	99.78	99.79	99.82

###  Model efficiency and real-time deployment feasibility

To ensure the OADL-IVIDC model is suitable for real-time use in vehicles, the model is assessed for its computational efficiency in terms of model size, inference time, and hardware usage. The model includes around 195,000 trainable parameters, making it lightweight compared to large models like Transformers, which often exceed 10 million parameters. On an NVIDIA Jetson Nano, a typical embedded automotive device, the model achieved an average inference time of 1.84 milliseconds per CAN message, allowing fast and timely intrusion detection within the vehicle’s control cycles. In terms of system resources, OADL-IVIDC used about 220 MB of RAM and reached a peak CPU usage of 42%, ensuring smooth operation without overloading the system. These results confirm that OADL-IVIDC is not only accurate but also efficient and practical for deployment in real-world, resource-constrained automotive environments.

## Conclusion and future work

This manuscript introduces the OADL-IVIDC framework for analyzing CAN messages with the primary goal of accurately detecting and classifying in-vehicle intrusions. The proposed approach begins with a data preprocessing stage that structures raw input data into an analyzable format. It employs an Attention-based Long Short-Term Memory (A-LSTM) model to capture temporal dependencies in the data, while performance is further enhanced through hyperparameter optimization using the RMSProp algorithm. The model’s effectiveness was validated using a publicly available car hacking dataset, demonstrating superior performance across multiple evaluation metrics. Future work may focus on improving the OADL-IVIDC framework by incorporating advanced feature selection and outlier detection techniques to further boost detection accuracy and computational efficiency. Additionally, to support real-time intrusion detection, the model can be extended to operate on live vehicular data streams, enabling dynamic threat identification during actual vehicle operation. Another important direction involves enhancing the scalability and adaptability of the model across different automotive platforms. Since vehicles often vary in their CAN architectures, message ID formats, and communication patterns, future enhancements could leverage transfer learning to fine-tune the model using minimal labeled data from new vehicle types, thereby reducing the overhead of full retraining and ensuring broader applicability.

## Data Availability

The data that support the findings of this study are openly available in [Car-Hacking Dataset for the intrusion detection] at https://ocslab.hksecurity.net/Datasets/car-hacking-dataset, reference number [21].
